# *Mycobacterium tuberculosis* Multidrug Resistant Strain M Induces an Altered Activation of Cytotoxic CD8^+^ T Cells

**DOI:** 10.1371/journal.pone.0097837

**Published:** 2014-05-16

**Authors:** Laura Geffner, Juan Ignacio Basile, Noemí Yokobori, Denise Kviatcovsky, Carmen Sabio y García, Viviana Ritacco, Beatriz López, María del Carmen Sasiain, Silvia de la Barrera

**Affiliations:** 1 Laboratorio de Inmunología de Enfermedades Respiratorias, Instituto de Medicina Experimental-CONICET, Academia Nacional de Medicina, Buenos Aires, Argentina; 2 Laboratorio de Micobacterias, Instituto Nacional de Enfermedades Infecciosas, ANLIS “Dr. Carlos G. Malbrán”, Buenos Aires, Argentina; Institute of Microbial Technology, India

## Abstract

In human tuberculosis (TB), CD8^+^ T cells contribute to host defense by the release of Th1 cytokines and the direct killing *of Mycobacterium tuberculosis* (*Mtb*)-infected macrophages via granule exocytosis pathway or the engagement of receptors on target cells. Previously we demonstrated that strain M, the most prevalent multidrug-resistant (MDR) *Mtb* strain in Argentine, is a weak inducer of IFN-γ and elicits a remarkably low CD8-dependent cytotoxic T cell activity (CTL). In contrast, the closely related strain 410, which caused a unique case of MDR-TB, elicits a CTL response similar to H37Rv. In this work we extend our previous study investigating some parameters that can account for this discrepancy. We evaluated the expressions of the lytic molecules perforin, granzyme B and granulysin and the chemokine CCL5 in CD8^+^ T cells as well as activation markers CD69 and CD25 and IL-2 expression in CD4^+^ and CD8^+^ T cells stimulated with strains H37Rv, M and 410. Our results demonstrate that M-stimulated CD8^+^ T cells from purified protein derivative positive healthy donors show low intracellular expression of perforin, granzyme B, granulysin and CCL5 together with an impaired ability to form conjugates with autologous M-pulsed macrophages. Besides, M induces low CD69 and IL-2 expression in CD4^+^ and CD8^+^ T cells, being CD69 and IL-2 expression closely associated. Furthermore, IL-2 addition enhanced perforin and granulysin expression as well as the degranulation marker CD107 in M-stimulated CD8^+^ T cells, making no differences with cells stimulated with strains H37Rv or 410. Thus, our results highlight the role of IL-2 in M-induced CTL activity that drives the proper activation of CD8^+^ T cells as well as CD4^+^ T cells collaboration.

## Introduction

Tuberculosis (TB) is still considered one of the main public health problems, with an estimated 8.7 million incident cases of TB in 2011 worldwide [1], being in Argentina the third cause of death by infectious diseases [2]. The up-surge of multidrug-resistant TB (MDR-TB) that is caused by *Mycobacterium tuberculosis* (*Mtb)* isolates resistant to at least the two most powerful anti-TB drugs, isoniazid (INH) and rifapim (RFP), are still a complication for TB eradication [3]. MDR-TB poses a real threat to TB control and elimination due to the alternative treatment that involves second line drugs, which are more expensive, more toxic and less effective, requiring longer treatment in MDR-TB patients to acquire a negative AFB sputum [4].

During 2003–2008, Argentina showed an average incidence of 142.3 cases of MDR-TB/year and 8.1 cases of XDR-TB/year being 75% of MDR-TB patients infected with strain M (both HIV positive and negative). This cluster belongs to the H2 subfamily, genotype SIT 2 [5] and was initially identified in a hospital outbreak in patients co-infected with HIV during the ‘90s [6]. In contrast, strain 410, a variant of strain M, was identified during the early epidemic as the cause of a single MDR-TB case that has remained unique despite the patient had being treated during 7 years in 3 different hospitals [7], suggesting that this strain has an impaired ability to cause disease in new hosts. As in epidemiology, a pathogen’s reproductive fitness is reflected in the number of secondary cases generated [8], M would have a higher fitness than the sporadic strain 410. Host immune response constitutes one of the more important evolutionary forces on *Mtb* evolution [9] so, it is conceivable that some of the differences in relative fitness among *Mtb* strains are due to a differential ability to evade the immune system. In this context, in human monocytes-derived macrophages (Mφ), strain M grows more slowly and elicits lower levels of TNF-α and IL-10 than strain 410, suggesting that strain M could remain rather unnoticed by the host Mφ [10]. On the other hand, both strains induce in vitro low IFNγ and similar IL-10 and IL-4 expression in T cells from healthy donors reactive to purified protein derivative (PPD) [11], but strain M induces higher IL-17 than strain 410 (Basile J, unpublished results), suggesting that both strains also differ in their ability to evoke memory T cell responses.

Cytotoxic T cell (CTL) activity has been associated with lysis of *Mtb*-infected Mφ [12], [13] and with reduction in *Mtb* viability [14], [15]. In experimental TB models, the role of CD8^+^ T cells in infection control has been demonstrated in mice [16], [17] and in macaques [18]. In patients with drug-susceptible TB [19], [20], [21] and MDR-TB [11] a weak *Mtb*-specific CTL response has been also shown. However, studies focused on T cell mediated lysis of Mφ infected with different *Mtb* strains are scarce. It has been recently demonstrated that virulence of *Mycobacterium* strains are associated with subverting CTL responses, thus contributing to early bacterial replication and subsequent persistence in the lungs [22]. In this line, we have previously shown that strain M in vitro elicits a remarkably low CD8-dependent CTL activity in terms of ability to lyse M-pulsed Mφ and expression of the degranulation marker CD107 [11]. Interestingly, the sporadic strain 410 induces a strong CTL response. So, the impaired CTL activity induced by M could be an evasion mechanism to avoid Mφ killing and also be related with its epidemiologic success. Hence, the aim of this work was to extend our previous findings and characterize M- and 410-induced CTL in terms of content of lytic molecules perforin, granzyme B and granulysin and CCL5 expression in CD8^+^ T cells as well as CD69 and CD25 activation markers and IL-2 expression in CD4^+^ and CD8^+^ T cells. Our results demonstrate that M-stimulated CD8^+^ T cells from PPD^+^ healthy donors show low content of lytic molecules and CCL5 expression together with an impaired ability to form conjugates with autologous M-pulsed Mφ. Besides, strain M induces low CD69 and IL-2 expression in CD4^+^ and CD8^+^ T cells. Also, low perforin, granulysin and CD107 expression induced by strain M in CD8^+^ T cells is recovered by addition of IL-2, suggesting that low M-induced CTL response could be a consequence of a deficient activation of CD8^+^ T cells.

## Materials and Methods

### Ethics Statement

This work was reviewed and approved by the Bioethics Committees from Academia Nacional de Medicina (Decision Number 23-03-2010) and Hospital Muñiz (DN 131-07, Project Number 145) and by the Teaching and Research Committee from Buenos Aires City government (DN 1217 2010) before the study began.

### Donors

Peripheral blood was obtained from BCG-vaccinated healthy donors recruited from laboratory personnel of the Academia Nacional de Medicina (n = 22, 12 women, 10 men; median age [percentiles 25–75] = 30 [27]–[46]). They were classified according to the reactivity to PPD in negative (naïve, PPD^neg^ N, n = 7) and positive (latent infection, PPD^+^ N, n = 15) All donors gave written informed consent as approved by the research ethics board of the institution. Exclusion criteria included a positive test for human immunodeficiency virus (HIV) and the presence of concurrent infectious diseases or noninfectious conditions like cancer, diabetes, or steroid therapy.

### Antigens

The two MDR strains from the Haarlem family M and 410 as well as the laboratory strain H37Rv, were grown in Middlebrook 7H9 broth (Difco Laboratories, Detroit, MI) at 37°C in 5% CO_2_ until log phase. Mycobacteria were harvested, sonicated to disrupt the clumps, washed thee times, and suspended in PBS free of pyrogens. Bacteria were killed by heating at 80°C for 1 h, suspended in PBS at an optical density at 600 nm of 1 (≈10^8^ bacteria/ml), and stored at −20°C until use. These mycobacterial suspensions contained soluble as well as particulate antigens.

### Mononuclear Cells

PBMC were isolated from heparinized blood by Ficoll-Triyosom gradient centrifugation and suspended in RPMI 1640 (HyClone; Thermo Scientific, UT) containing 100 U/ml penicillin, 100 µg/ml streptomycin and 10% heat-inactivated fetal calf serum (FCS, Invitrogen, Gibco) (complete RPMI medium).

### Macrophage Isolation and Culture

Adherent cells (85–95% monocytes) were obtained from PBMC by plastic adherence. PBMC (5×10^5^ cells/well) were plated at the bottom of 24 well Falcon plates for 2 h at 37°C. After removing the non-adherent cells, monocytes were washed with warm RPMI medium and then cultured in complete RPMI medium at 37°C in a humidified 5% CO_2_ atmosphere for 6 days (d). On 5 d of incubation, Mφ were stimulated with *Mtb* strains for further 18 h (1×10^6^ bacteria/ml). Mφ kept under the same conditions but without addition of antigen were used as controls.

### PBMC Cultures

PBMC (2×10^6^ cells/ml) were cultured for 18 h or 6 d in Falcon polystyrene tubes (BD Bioscience, San Jose, CA, USA) at 37°C in a humidified 5% CO_2_ atmosphere, in complete medium with or without strains M, 410, or H37Rv (2∶1 ratio of *Mtb* to PBMC). In some experiments, recombinant Interleukin-2 (Biolegend Inc., San Diego, CA, USA) or neutralizing anti-human IL-2 monoclonal antibody (10 µg/ml, clone MQ1-17H12, BD Bioscience, NJ, USA ) was added at the onset of PBMC culture. In some experiments, PBMC were cultured for 1 h alone or with strain M and then cultured with or without anti-CD3 (10 µg/ml) and/or anti-CD28 (5 µg/ml) (eBioscience, San Diego, CA, USA) for further 18 h. In parallel, PBMC were simultaneously cultured for 18 h with anti-CD3 and/or CD28 and *Mtb* strains.

### Isolation of CD8^+^ T Cells

CD8^+^ T cells were isolated from PBMC cultured for 6 d alone or with strain H37Rv, M or 410 by negative selection. Briefly, PBMC were treated first with anti-human TCR Pan gamma delta (mouse IgG1, clone IMMU510, Beckman Coulter Co, CA, USA), anti-human CD56 (mouse IgG1, κ, clone B159, BD Bioscience), anti-human CD4 (mouse IgG 1,κ, clone QS4120, Ancell Corporation, Bayport, Mn, USA), anti-human CD14 (mouse Ms IgG2a, κ, clone M5E2, BD Biosciences) and anti-human CD19 (mouse IgG1, κ, clone HIB19, BD Bioscience) monoclonal antibodies for 30 min and then with goat anti-mouse IgG-coated beads (Dynabeads, Life Technologies Corporation, CA, USA) for further 30 min at 4°C. Non rosetted cells were recovered employing a magnet.

### Immunofluorescence Analysis

#### Surface membrane expression

The following monoclonal antibodies (MoAbs) were used to evaluate surface marker expression in ex vivo or cultured PBMC and macrophages: PE-Cy5 or PE conjugated anti-CD8 and anti-CD3, FITC-anti-CD195 (CCR5), PE-anti-CD69, PE-anti-CD25 and PerCP-Cy5-anti-CD14 (BD Bioscience), PE- or FITC-conjugated anti-CD11a (LFA-1), PE-anti-CD54 (ICAM-1), FITC-anti-CD45RA, PE-anti-CD62L and FITC-anti-CD54 (eBioscience).

#### Intracellular expression of lytic molecules and RANTES in CD8+T cells

Intracellular perforin, granzymes B, granulysin and CCL5 were determined in 5-d PBMC cultures. Cells were surface stained with anti-CD8 and anti-CD3 and then fixed with 0.5% paraformaldehyde and permeabilized with FACS solution 2 (BD Bioscience) before addition of FITC-anti-perforin, PE-anti-granzyme B or PE-anti-granulysin MoAbs (eBioscience).

#### Intracellular cytokine expression

Intracellular IL-2 expression was evaluated in 5-d PBMC cultures. Briefly, Brefeldin A (5 µg/ml; Sigma Chemical Co., St. Louis, MO) was added for the last 4 h of culture to block cytokine secretion, and cells were surface stained with anti-CD4, anti-CD8 and anti-CD3 fixed and permeabilized as indicated above before addition of PE-anti-IL-2 (BD Bioscience).

#### CD107 surface expression

CD107a/b lysosome-associated membrane protein-1/2 expression was used to measure CD8^+^ T lymphocyte degranulation, as previously described [23]. Briefly, 5-d PBMC cultures (2×10^6^ cells/ml in complete medium) stimulated or not with *Mtb* strains (2∶1 ratio of *Mtb* to PBMC) were incubated with FITC-anti-CD107 MoAb (BD Pharmingen) for further 4 h at 37°C in a 5% CO_2_ incubator. After that, cells were washed and stained for CD3 and CD8 expression.

#### CD8 T cell- Mφ conjugates formation

The ability of *Mtb*-stimulated CD8^+^ T cells to bind to *Mtb*-pulsed Mφ was determined by flow cytometry (FACS) as previously described [24]. 5-d cultured Mφ were detached from culture plates by gentle pipetting with ice-cold RPMI medium, washed, suspended in PBS-0.1% BSA at 2.5×10^6^ cells/ml and incubated with 2 µM CFSE (Invitrogen, USA) for 30 min at 37°C. The reaction was stopped by the addition of an equal volume of FCS, followed by 2 min incubation at room temperature. After two washes, CFSE-stained Mφ were suspended in complete medium (2.5×10^6^ cells/ml) and stimulated with *Mtb* strains (2∶1 bacilli to Mφ ratio) for 2 h in polystyrene tubes, cells were then washed and suspended in complete medium for conjugate formation assay. In parallel, PBMC cultured alone or with *Mtb* strains for 5 d were stained with PECy5-anti-CD8 or the corresponding isotype matched MoAb for 30 min, washed and suspended in complete medium at 1×10^7^ cells/ml. Afterward, CD8-stained PBMC were mixed with 2.5×10^5^ CFSE^+^ Mφ in a 10∶1 ratio at a final volume of 0.2 ml and incubated at 37°C for 1 h in the presence or not of neutralizing anti-CCL5 antibody (10 µg/ml, clone 2D5, IgG1,κ, BD Bioscience). Nonspecific conjugates were dispersed by gentle vortexing and cells were fixed with 4% paraformaldehyde and incubated for further 15 min at 4°C. Finally, cells were washed and gently suspended in Isoflow (BD Biosciences) for FACS analysis. The following controls were made: Mφ alone, PBMC alone stained with anti-CD8 or the corresponding isotype and Mφ plus PBMC cultured without antigen.

In all cases, lymphocyte or Mφ gates were set according to forward and side-scatter parameters, excluding cell debris and apoptotic cells. Stained cells were analyzed by FACS. Twenty thousand events were acquired for each cell preparation, using a FACSCan flow cytometer (BD Bioscience) with CellQuest. FCS Express software (De Novo Software, Los Angeles, CA) was used for the analysis. Results were expressed as: a) percentages of positive cells within CD8^+^ T cells, b) percentages of positive cells within the Mφ population for surface or intracellular analysis, and c) the percentage of CFSE^+^CD8^+^ cells within the Mφ population for conjugate formation.

### Evaluation of CD8 T Cell- Mφ Conjugates Formation by Fluorescence Microscopy

Control and *Mtb*-stimulated CD8^+^ T cells were stained with CFSE as described in materials and methods and cultured with 2.5×10^5^ autologous non-stimulated or *Mtb*-stimulated Mφ in a 5 CD8 to1 Mφ ratio (final volume of 0.2 ml) during 1 h at 37°C. Cells suspensions were fixed with 4% paraformaldehyde (in ice, 10 min), permeabilized with Triton X-100 (15 min) and stained with TRICT-anti-phalloidin (15 min). Finally, cellular conjugates were qualitatively and quantitatively evaluated by epifluorescence microscopy in cytospin preparations, using 4,6-diamidino-2-phenylindole (DAPI) as counter-stain (Molecular Probes). Conjugates formation was detected among CD8^+^ T cells (green cells) and Mφ (red cells) and the percentage of CD8^+^ T cells adhered to 300 Mφ was calculated.

### Statistical Analysis

Data were expressed as medians and 25th to 75th percentiles. The Friedman test was performed to compare responses to different treatments within each group, followed by the Wilcoxon test. Correlation between parameters was analyzed by Spearman test and the linear regression line test. All statistical analyses were two sided, and the significance level adopted was for *P* values of <0.05. The analysis was performed using the statistical software SPSS 15.0 for Windows (SPSS Inc., IL) and Graphpad Prism 4.0 (Graphpad Software Inc., CA).

## Results

### M-induced CD8^+^ T Cells Displayed a Low CD8-macrophage Conjugate Formation

**Figure 1:**
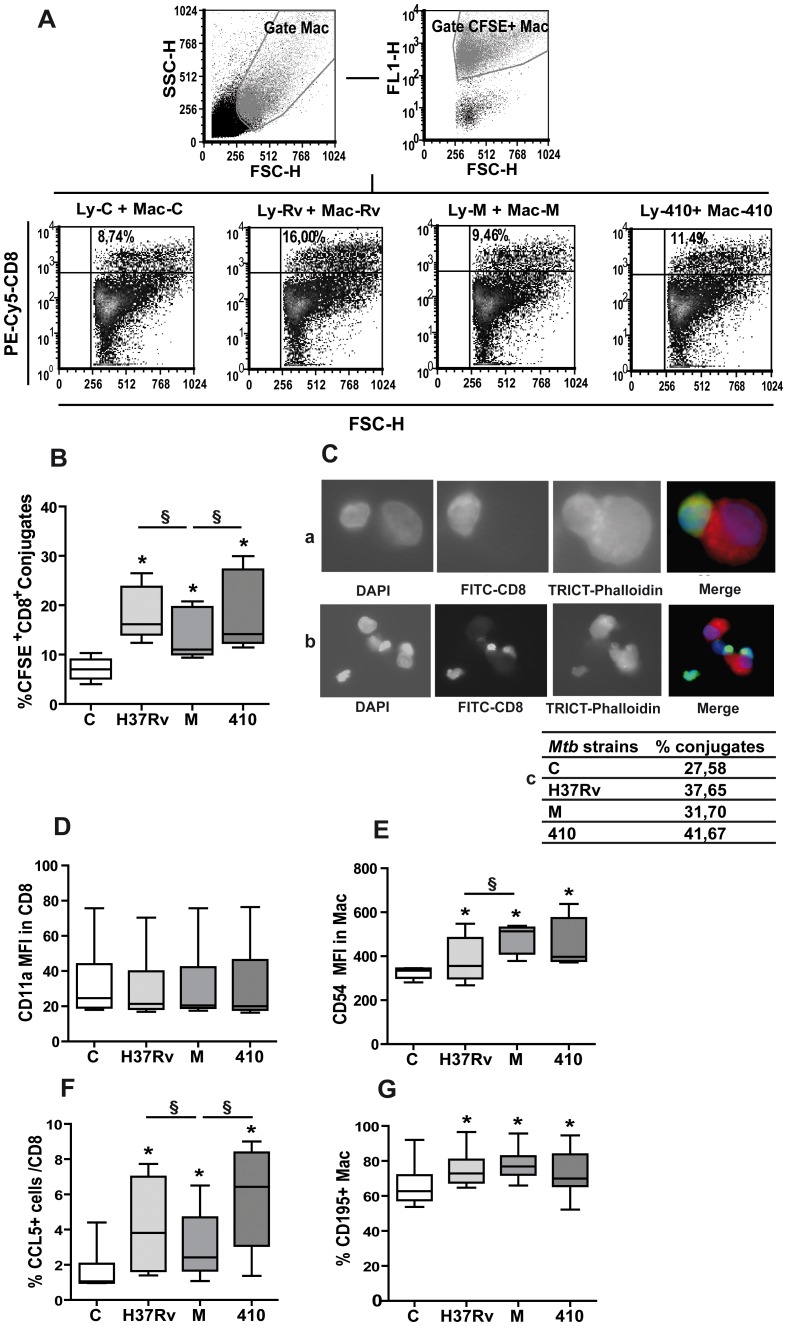
M-induced CD8^+^ T cells display a low CD8-macrophage conjugate formation. **A–C.** Conjugates between CD8^+^ T cells and macrophages. PBMC from 7 PPD^+^ healthy donors (N) were cultured for 6 d in absence (control, C) or presence of strains H37Rv, M or 410 (*Mtb* to PBMC ratio = 2∶1). Upon CD8-staining, they were mixed with autologous CFSE-stained macrophages (Mφ) pulsed or not (Mac-C) with *Mtb* strains (*Mtb*: Mφ ratio = 2∶1) for 2 h Then cell suspensions were analyzed FACS. Results are expressed as percentage (%) of CD8^+^ T cells in the CFSE^+^-Mφ gate. Representative dot plots (**A**) and cumulative graphs (**B**) (medians and percentiles 25–75) are shown. Statistical differences: PBMC+*Mtb* strains vs. control PBMC: * = p<0.05, between strains: § = p<0.05. **C.** CD8^+^ T cells isolated from 6 d-cultured PBMC were stained with CFSE and cultured with autologous non-stimulated or *Mtb*-stimulated Mφ (5 CD8 to 1 Mφ ratio) for 1 h at 37°C. Cells suspensions were fixed with 4% paraformaldehyde, permeabilized with Triton X-100 (15 min) and stained with TRICT-anti-phalloidin. Finally, cellular conjugates were qualitatively and quantitatively evaluated by epifluorescence microscopy in cytospin preparations, using 4,6-diamidino-2-phenylindole (DAPI) as counter-stain. Conjugates formation was detected among one (a) or more (b) CD8^+^ T cells (green cells) and Mφ (red cells) and % of CD8^+^ T cells adhered to 300 Mφ was calculated; representative data are shown in the embedded table (c). **D.** Surface CD11a expression in CD8^+^ T cells from control or *Mtb*-stimulated PBMC. Results are expressed as median fluorescence intensity (MFI) within the CD3^+^CD8^+^ lymphocyte gate (n = 12, medians and percentiles 25–75). **E.** Surface CD54 expression in Mφ cultured alone (control, C) or with *Mtb* strains for 18 h Results are expressed as MFI (medians and percentiles 25–75, n = 6) in the macrophage gate. **F.** Intracellular CCL5 expression in control and *Mtb*-stimulated CD3^+^CD8^+^ lymphocytes. Results are expressed as % CCL5^+^ cells within the CD3^+^CD8^+^ lymphocyte gate (medians and percentiles 25–75, n = 15). **G.** Surface CCR5 expression in control and *Mtb*-stimulated Mφ. Results are expressed as % CCR5^+^ cells in the Mφ gate (medians and percentiles 25–75, n = 6). In all cases, statistical differences: PBMC+*Mtb* vs. control cells: * = p<0.05, § = p<0.05.

Activation and degranulation require the development of an immunological synapse evidenced by the formation of conjugates between CTL and antigen presenting cell (APC) [25]. So, we determined the ability of CD3^+^CD8^+^ T cells stimulated with strains M or 410 to form conjugates with CFSE-labeled *Mtb-*pulsed-Mφ by FACS. Strain H37Rv was also included as control. Results showed that M induced lower percentage of CD8^+^-CFSE^+^ conjugates than strain 410 and H37Rv in PPD^+^ N (Figure 1A and B), and these results were confirmed by fluorescence microscopy (Figure 1C). Considering that antigen recognition in the context of MHC molecules by target cells is the main step of CTL–target cell conjugate formation, the expression of surface HLA-abc was evaluated on Mφ, observing that *Mtb* strains did not modify its expression (data not shown). Thus, lack of conjugate formation by M-stimulated cells could not be ascribed to an impaired of MHC class I molecule expression on M-stimulated Mφ. As antigen-specific cellular unions are stabilized by adhesion molecules [25], and CD11a/CD54-mediated adhesion has been established as a critical event strengthening Mφ-CD8 T cell contact leading to an optimal cytotoxic response [26], the expression of CD11a in CD8^+^ T cells from 5 d-*Mtb* stimulated PBMC and of CD54 in 18 h pulsed Mφ were evaluated. As shown in Figure 1D, no differences in CD11a expression in CD8^+^T cells from PPD^+^ N were observed among *Mtb* strains. Regarding CD54 in Mφ, M induced higher expression than H37Rv but similar to 410 (Figure 1E), suggesting that the less conjugate formation by strain M does not relay on CD11a/CD54 molecules.

It is known that Mφ infected with virulent *Mtb* trigger CCL5 expression in CD8^+^ T cells, being this chemokine necessary to attract *Mtb*-infected Mφ before killing them though CTL response [27]. Thus, CCL5 expression in CD8^+^ T cells from 5 d cultured PBMC and its receptor CD195 in 18 h-pulsed Mφ, were evaluated. Strain M induced a lower percentage of CCL5^+^ cells in CD8^+^ T cells from PPD^+^ N than H37Rv or 410 (Figure 1F), but no differences were observed in the percentage of CD195^+^ Mφ (Figure 1G). Also, in accordance with literature [27], most CCL5^+^CD8^+^ T cells expressed surface CD107 and no differences were observed among strains (data not shown). Furthermore, neutralization of CCL5 diminished Mφ-T conjugate formation by *Mtb*-stimulated CD8^+^ T cells and was not dependent on the strain (% CD8^+^/CFSE^+^ conjugates: H37Rv = 18.9 (14.8–26.4), H37Rv+a-CCL5 = 4.1 (3.5–5.3) p<0.05; M = 13.2 (11.1-16-3), M +a-CCL5 = 3.2 (2.5–4.8), p<0.05; 410 = 16.7 (13.6–26.6), 410+ a-CCL5 = 3.8 (2.8–5.2, n = 6). Consequently, the low conjugate formation and low degranulation observed by strain M could be explained by the low CCL5 expression.

### M-stimulated CD8^+^ T Cells Express Low Levels of Lytic Molecules

**Figure 2:**
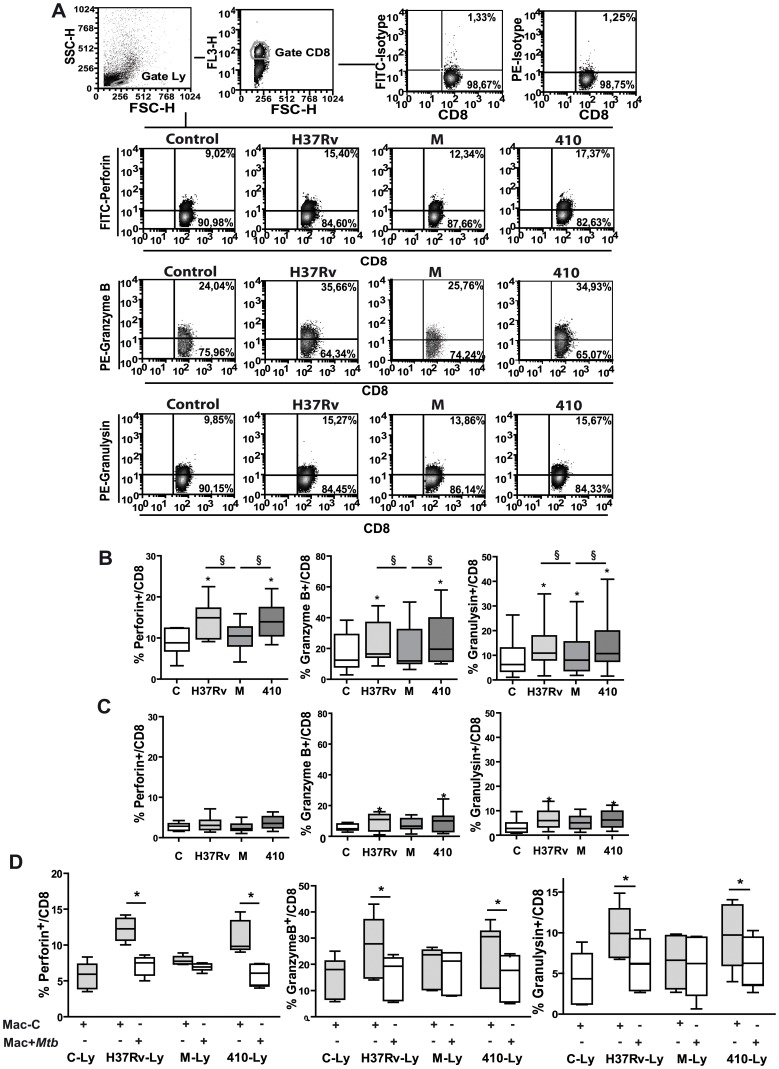
M-stimulated CD8^+^ T cells express low levels of lytic molecules. Intracellular expression of perforin, granzyme and granulysin in control or *Mtb-*stimulated CD8^+^ T cells from 15 PPD^+^ N (**A and B**) and 7 PPD^NEG^ N (**C**). Results are expressed as % of Perforin^+^, of Granzyme B^+^ or of Granulysin^+^ in the CD3^+^CD8^+^ T lymphocyte gate. **A** Representative dot plots; **B and C** graphs showing %Perforin^+^/CD8, %Granzyme B^+^/CD8 and %Granulysin^+^/CD8 (median and percentiles 25–75). Statistical differences: PBMC+*Mtb* vs. control PBMC * = p<0.05, between strains: § = p<0.05. **D.** PBMC from 5 PPD^+^ N were cultured alone (Control Ly) or with *Mtb* strains (H37Rv-Ly, M-Ly and 410-Ly) for 6 d and then co-cultured for further 4 h with non-stimulated (Ly+control-Mac) or *Mtb*-stimulated (Ly+*Mtb*-Mac) autologous macrophages. Cells were surface stained for CD3 and CD8 and intracellular perforin expression and evaluated by FACS. %Perforin^+^/CD8, %Granzyme B^+^/CD8 and %Granulysin^+^/CD8 was then determined and results are expressed as (median and percentiles 25–75). Statistical differences Ly+Mac+*Mtb* vs Ly+Mac-C: * p<0.05.

To determine the profile of cytotoxic molecules, the intracellular expression of perforin, granzyme B and granulysin was evaluated in CD8^+^ T cells in 5 d-PBMC cultures stimulated with strains H37Rv, M or 410. As shown in Figure 2A and B, all *Mtb* strains enhanced the percentage of CD8^+^ T cells expressing perforin, granzyme B and granulysin in PPD^+^ N; however, strain M was a lower inducer of these molecules compared to strains H37Rv or 410. As a negligible enhancement in the expression of lytic molecules on CD8^+^ T cells from PPD^NEG^ N in response to *Mtb* was observed (Figure 2C), we can assume that this effect is antigen specific.

Activated CD8^+^ T cells loss perforin expression during the degranulation process [23]; thus we evaluated the expression of lytic molecules in 6 d-cultured CD8^+^ T cells from PPD^+^ N exposed for further 6 h with autologous Mφ. *Mtb*-stimulated Mφ induced a loss of CD8^+^ T cells expressing lytic molecules in H37Rv- and 410-stimulated PBMC, while non-stimulated Mφ did not (Figures S1–S3 and Figure 2D). On the contrary, a low or negligible reduction was observed in M-stimulated cells. Thus, low content of lytic molecules and low granule exocytosis in CD8^+^ T cells could be involved in the impaired M-induced CTL response.

### M Strain Induces Low Expression of CD69 in CD8^+^ T Cells

**Figure 3:**
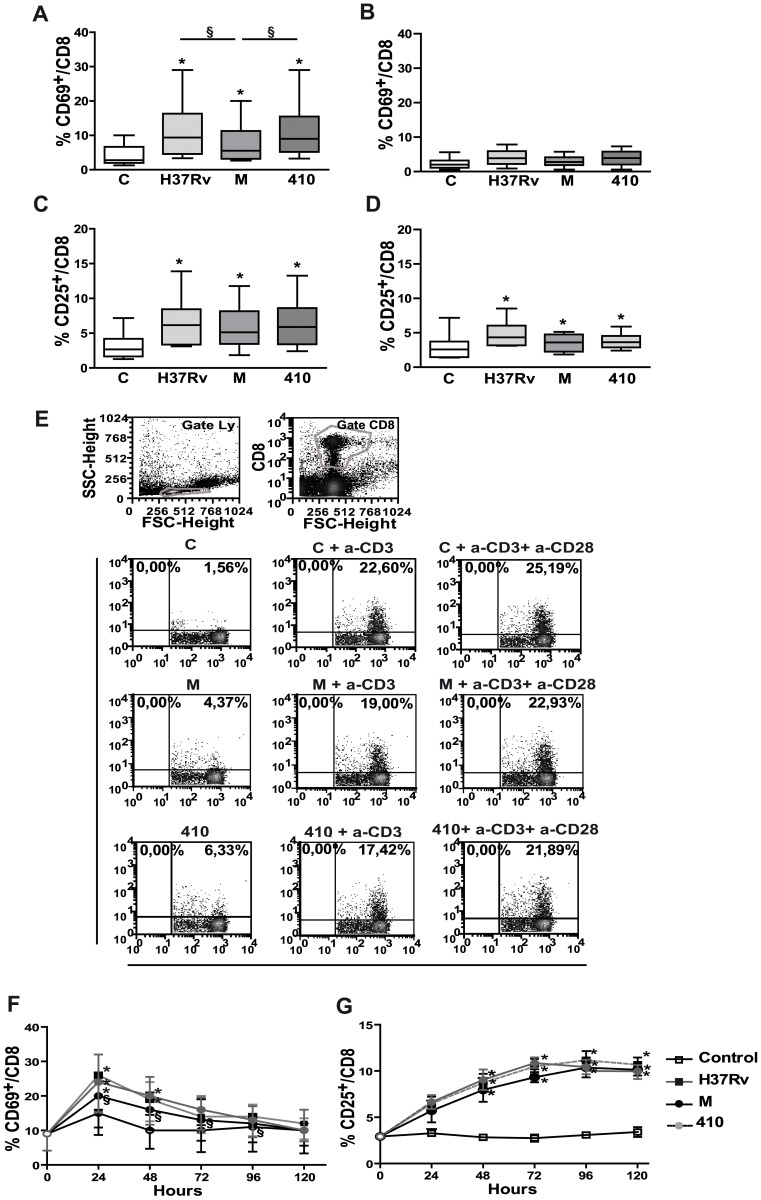
M strain induces low expression of CD69 in CD8^+^ T cells. **A and B,** Surface CD69 expression on CD8^+^ T cells from 18 h-cultured PBMC in 15 PPD^+^ N (A) and 7 PPD^NEG^ N (B). **C and D,** Surface CD25 expression in CD8^+^ T cells from 5 days-cultured PBMC in 13 PPD^+^N (C) and 7 PPD^NEG^ N (D). Results are expressed as %CD69^+^ and %CD25^+^ cells in the CD8^+^CD3^+^ T cells gate. Statistical differences: PBMC+*Mtb* strains vs. control PBMC * = p<0.05, among strains: § = p<0.05. **E.** PBMC from PPD^+^ N (n = 3) were cultured for 18 h alone (C) or with strains H37Rv, M or 410 with or without anti-CD3 and/or anti-CD28. Representative dot plots show the % of CD69^+^ cells in the CD3^+^CD8^+^ lymphocyte gate. **F and G.** PBMC from 6 PPD^+^ N were cultured alone or with *Mtb* strains and the %CD69^+^ (F) and %CD25^+^ (G) cells within the CD3^+^CD8^+^ lymphocyte gate was determined at different time points. Statistical differences, PBMC+*Mtb* strains vs. control PBMC: * = p<0.05, between strains: § = p<0.05.

Expression of lytic molecules can be down-regulated by differentiation and maturation [24], [28] or up-regulated by activation [29] of CD8^+^T cells. Therefore, expansion of memory CD8^+^ T cells as well as early activation marker CD69 and late activation marker CD25 were measured in CD8^+^ T cells at different time points upon *Mtb* stimulation. Not evidence of a differential expansion of memory CD8^+^ T cells was observed by *Mtb* strains (data not shown). Regarding activation markers, *Mtb* strains induced CD69 expression in cells from PPD^+^N, but strain M induced the lowest (Figure 3A). In addition, not significant differences in CD25 expression was detected among strains (Figure 3B). Although the same pattern in the expression of CD69 and CD25 was observed in CD8^+^ T cells from PPD^NEG^ N, their proportion were notably diminished comparing to PPD^+^ N (Figure 3C and D).

To determine if an active inhibition mechanism was involved in M-induced T cell activation, PBMC were pre-incubated for 1 h with *Mtb* strains and then activated via TCR with anti-CD3 with or without anti-CD28 for 18 h Our results showed that *Mtb* strains did not inhibit TCR signaling at least in terms of CD69 expression (data nor shown). However, if *Mtb* strains were present the entire 18 h upon CD3/CD28 stimulation, a slight decrease in CD8^+^CD69^+^ cells was observed and it was not dependent on the strains (Figure 3E). In contrast CD25 expression was not modified (data not shown). Given that CD4^+^ and CD8^+^ T cells stimulated with mycobacterial antigens show delayed kinetic in CD69 and CD25 expression compared to PHA stimulation in PPD^+^ N [30], we wondered if the low CD69 expression could be due to a delay in CD8^+^ T cells activation. Thus, the kinetics of CD69 and CD25 expression (1–5 d) in PBMC cultured with *Mtb* strains were determined. Our results showed that the highest CD69 expression was observed upon stimulation for 24 h with the three strains, though strain M induced the lowest expression between 24–96 h (Figure 3F). Furthermore, the peak of CD25 expression was not dependent on the strain and it was observed upon 72 h of stimulation (Figure 3G).

### IL-2 Expression is Impaired in M-stimulated CD8^+^ T Cells

**Figure 4:**
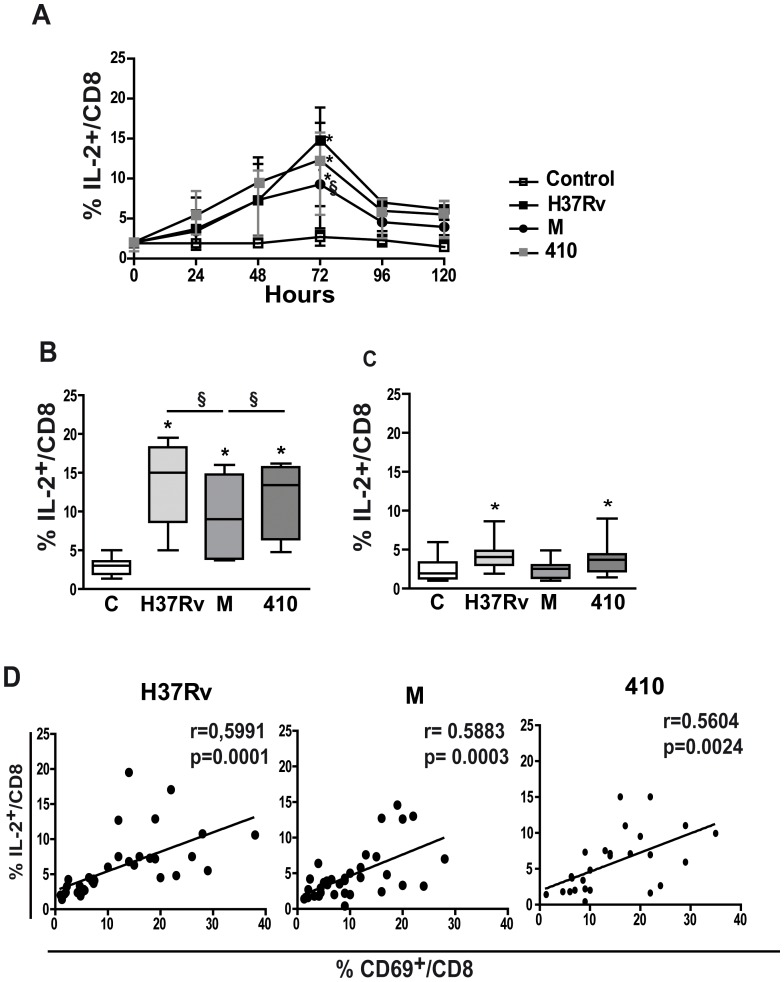
M induces low IL-2 expression in CD8^+^ T cells. PBMC from PPD^+^ N were cultured alone or with H37Rv, M or 410 strains for 24–120 h and then intracellular IL-2 expression was determined in CD8^+^ T cells. Results are expressed as % IL-2^+^ cells within the CD3^+^CD8^+^ lymphocyte gate (%IL-2^+^/CD8^+^) (median and 25–75 percentiles). **A.** % IL-2^+^/CD8^+^ at different time points upon antigen stimulation (n = 6). Statistical analysis, PBMC+*Mtb* strains vs. control PBMC * = p<0.05, between strains: § = p<0.05. **B and C.** %IL-2^+^/CD8^+^ at 72 h cultured PBMC from 8 PPD^+^ N (B) and 7 PPD^NEG^ N (C). Statistical differences: PBMC+*Mtb* strains vs. control PBMC * = p<0.05, between strains: § = p<0.05. **D.** PBMC from PPD^+^ N (n = 5) were stimulated with H37Rv, M or 410 and the %CD69^+^ and %IL-2^+^ cells determined in CD3^+^CD8^+^ cells at different time points. Correlation between %IL-2^+^ and %CD69^+^ cells in CD8^+^ T cells was calculated considering all time points for each *Mtb* strain. Data was analyzed using the linear regression test with a significance level of p<0.05.

As IL-2 modulates CD8^+^ T cells differentiation/expansion upon antigen stimulation [31], its intracellular expression in *Mtb-*stimulated CD8^+^ T cells from PPD^+^ N was evaluated. As shown in Figure 4A, IL-2 expression in CD8^+^ T cells was induced by all strains being the highest expression at 72 h; however, M showed the lowest percentage at this time point (Figure 4B). In the same line, IL-2 expression was poorly induced in cells from PPD^NEG^ N upon *Mtb* stimulation (Figure 4C) and, furthermore, they showed the lowest IL-2 levels compared to PPD^+^ N (p<0.05 for all strains). Considering that CD69 is a regulator of the immune response that induces IL-2 expression [32], we wondered if an association between CD69 and IL-2 expressions in CD8^+^ T cells could be established. As can be observed in Figure 4D, a direct association between CD69 and IL-2 expressions was detected for the three strains suggesting that IL-2 expression is closely dependent on early T cell activation.

### M Strain Induces a Deficient CD4^+^ T Cell Activation with Low IL-2 Expression

**Figure 5:**
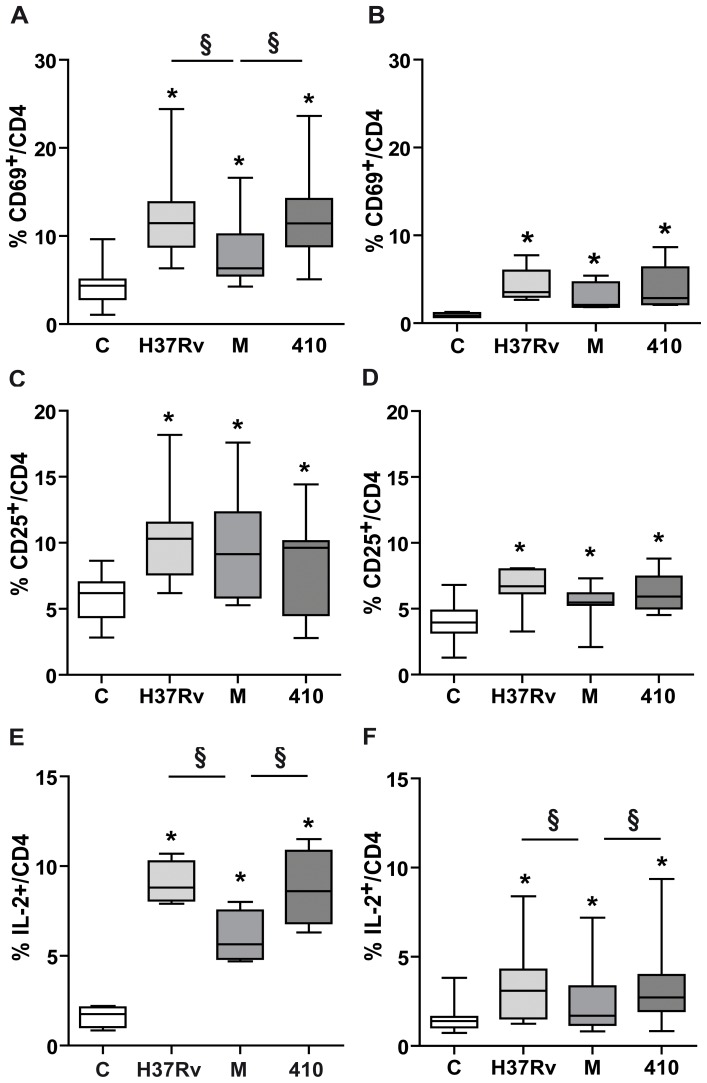
M strain induces a deficient CD4^+^ T cell activation with low IL-2 expression. PBMC from 11 PPD^+^ N (A, C and E) and 7 PPD^NEG^ N (B, D and F) were cultured for 72 h alone or with *Mtb* strains H37Rv, M or 410 and then tested for surface expression of CD69 (A and B) and CD25 (C and D) and intracellular expression of IL-2 (E and F). Results are expressed as % of positive cells in the CD3^+^CD4^+^ lymphocyte gate. Data are shown as median and 25–75 percentiles. Statistical differences: PBMC+*Mtb* strains vs. control PBMC * = p<0.05, between strains: § = p<0.05.

CD8^+^ T lymphocytes depend on cytokines produced by activated CD4^+^ T cells to sustain expansion of the response and acquisition of effector/memory traits, mainly IL-2 and IL-21 [31]. Therefore, IL-2, CD69 and CD25 expressions were determined in CD4^+^ T cells at their optimal time of expressions. As shown in Figure 5A, M induced low percentage of CD69^+^ cells from PPD^+^ N while there were not differences in the percentage of CD25^+^ cells among strains (Figure 5B). IL-2 was induced by all *Mtb* strains, thought strain M induced the lowest proportion of IL-2^+^/CD4^+^ T cells (Figure 5C). The same tendency was observed in CD4^+^ T cells from PPD^NEG^ N but all these markers showed lower levels than in PPD^+^ N (p<0.05 for all strains) (Figures 5B, D and F). Taking together, these results suggest that strain M would be impairing the collaboration from CD4^+^ T cells to CD8^+^ T cells.

### Added Exogenous IL-2 Restores Perforin and CD107 Expression in M-stimulated CD8^+^ T Cells

**Figure 6:**
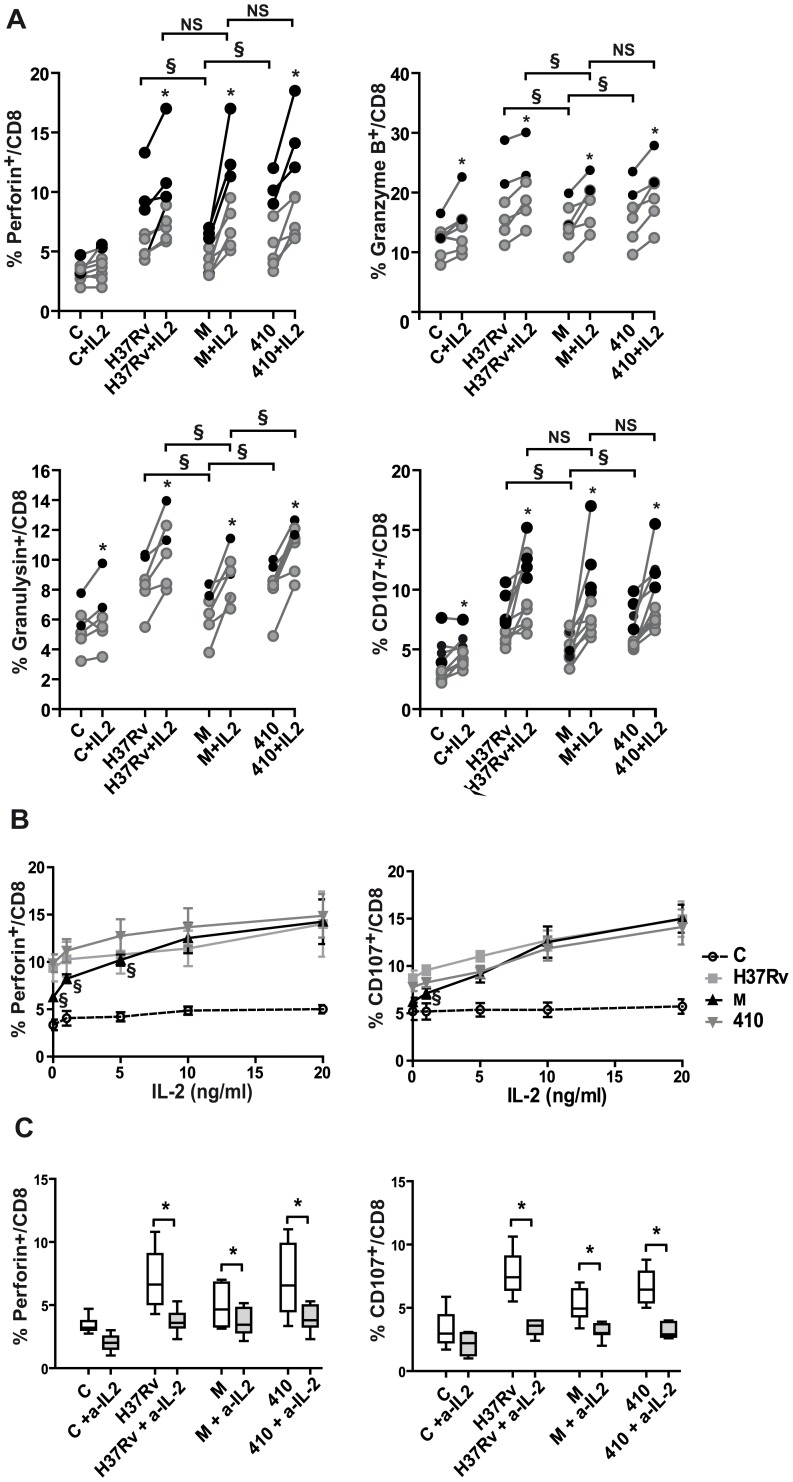
IL-2 enhances CD107 expression in CD8^+^ T cells. **A.** PBMC from 12 PPD^+^ N were cultured for 5 d alone or with *Mtb* strains H37Rv, M or 410 in the presence or not of IL-2 (100 U/ml). Then intracellular expression of perforin, granzyme B and granulysin and surface expression of CD107 were determined on CD8^+^ T cells. Results are expressed as % of positive cells in the CD3^+^CD8^+^ T cells gate and individual data are shown (Black dots = PPD+ N; white dots = PPD^NEG^ N). Statistical differences: PBMC+IL-2 vs. control PBMC+IL-2 * = p<0.05, between strains: § = p<0.05. B. PBMC from 10 PPD^+^N were cultured for 5 d with *Mtb* strains with the addition or not of different amounts of IL-2. Then the % perforin^+^ and %CD107^+^ cells were determined in CD3^+^CD8^+^ T cells and dose response curves are shown. Statistical differences PBMC+IL-2 vs. non treated cells: * = p<0.05. **C.** PBMC from 5 healthy donors were cultured for 5 d with *Mtb* strains in the presence or not of neutralizing anti-IL-2. Then the % perforin^+^ and %CD107^+^ cells were determined in CD3^+^CD8^+^ T cells. Results are expressed as mean and 25–75 percentiles. Statistical differences PBMC+a-IL-2 vs. untreated cells: * = p<0.05.

So far our results suggest that IL-2 seems to be involved in CTL activity, thus the effect of adding IL-2 at the onset of PBMC cultures was assessed. As shown in Figure 6A, the percentage of perforin^+^, granzyme^+^ and granulysin^+^ in *Mtb*-stimulated-CD8^+^T cells increased by addition of IL-2. While IL-2 addition enhanced perforin^+^ CD8^+^ T cells making not differences among the strains, granzyme B^+^ and granulysin^+^ cells induced by strain M remained at lower levels than strains H37Rv and 410. In addition, IL-2 also increased spontaneous and *Mtb*-induced CD107 expression (p<0.05 for all *Mtb* strains), and differences among the strains were not found. Furthermore, enhancement of CD107^+^ and perforin^+^ expression was dose-dependent for all strains (Figure 6B) while IL-2 neutralization abrogated their expression (Figure 6C). Thus, these results suggest that lack of IL-2 is involved in the low CTL response induced by M.

## Discussion

The control of human TB depends on the effective killing of infected Mφ by activated CD8^+^ T cells by the coordinated interaction of chemotactic and granule-associated effector molecules. In this study we demonstrated that the low CTL activity developed by strain M is due to its poor capacity to induce Mφ-CD8^+^T cell conjugates formation, lytic molecules and CCL5 in CD8^+^ T cells from PPD+ N. In addition, strain M also induced low CD69 and IL-2 expression in CD4^+^ and CD8^+^ T cells. Besides, the addition of IL-2 enhanced perforin, granulysin and CD107 expression in M-stimulated CD8^+^ T cells.

The interaction between CTLs and their target cells involves antigen recognition, effector–target cell binding, and release of cytotoxic granule content. Both, activation and lytic molecule degranulation, require the direct contact between CTL and Mφ with the development of an immunological synapse evidenced by the formation of conjugates [25]. Herein, we observed that Mφ-CD8 conjugate formation by strain M was lower than with strains H37Rv and 410. The low conjugate formation could not be ascribed to differences in the expression of recognition or adhesion molecules because similar expression of CD11a on CD8^+^ T cells as well as CD54 and MHC class I molecules on Mφ were observed for the three *Mtb* strains. However, we found that M induced lower CCL5 expression in CD8^+^ T cells than strains 410 and H37Rv but similar CD195 upregulation on Mφ. Since CCL5 released by CD8^+^ T cells provides a host mechanism to attract H37Rv-infected Mφ via CD195 and kill the pathogen intracellularly [27], the decreased CCL5 expression in M-stimulated CD8^+^ T cells could result in impaired Mφ attraction and low conjugate formation.

CTLs store cytolytic and antimicrobial molecules like perforin, granzyme and granulysin respectively, which are released from the secretory granules into the intercellular space between the CTL and the infected target cells [33]. Perforin forms pores in cellular membranes to facilitate entry of granzymes and/or granulysin to the intracellular compartments [34], [35]. Once inside the target cell, granzymes A and B induce apoptosis [36], [37] while granulysin, a member of the saponin-like family, can trigger apoptosis of the infected cell and directly attack mycobacteria inside the endosomes of infected Mφ reducing *Mtb* growth [38]. In this context, whereas the development of chonic pulmonary TB is associated with reduced perforin and granulysin expression in CD8^+^ T cells at the infection site that is consistent with a reduced CD8^+^ T cell maturation, an increase of these lytic molecules is associated with bacteria growth control [39]. Immediate CCL5 secretion [40] as well as perforin and granzymes upregulation [41] has been described to occur upon CD8^+^ T cells stimulation; thereby, deficient *up*-regulation of lytic molecules in CD8^+^ T cells could be the consequence of deficient CD8^+^ T cell activation. Herein we showed that strain M induced a poor *up*-regulation of perforin, granzyme B and granulysin expression as well as low percentages of CD69 in CD8^+^ T cells. Thus, low expression of lytic effector molecules together with impaired CCL5-mediated attraction could account for the impaired lysis of M-stimulated Mφ observed in healthy donors and TB patients [11]. Furthermore, being CD69 considered not only an early activation marker but also a marker of cytotoxic activity in NK cells and certain T cell clones [42], its low expression in CD8^+^ T cells suggests that the poor M-induced CTL activity involves a deficient activation of effector cells.

A direct inhibition by *Mtb* on murine CD4^+^ T cells activation has been demonstrated [43], while *M. leprae* inhibits CD69 and CD25 expression, proliferation and IL-2 production in human PBMC [44]. In contrast to these studies, we showed that strain M did not inhibit TCR signaling and CD69 expression in CD8^+^ T cells even when this strain was present along the entire culture. In addition, we found no differences in the kinetic of CD69 expression, suggesting that its low expression is not due to a delayed CD8^+^ T cells activation caused by strain M, as it was observed with other mycobacterial antigens [30]; however, we can not rule out the involvement of other co-stimulatory molecules. Signaling trough CD69 controls IL-2 and IFN-γ gene expression at the transcriptional levels [45], [46] as well as Stat-5 mediated IL-2 signaling. As we found a direct correlation in the percentages of CD69^+^CD8^+^ and IL-2^+^CD8^+^ T cells, the low autocrine IL-2 expression induced by strain M could be attributed to an unsuccessful CD69 signaling. Furthermore, CD4^+^ T cells also showed an ineffective activation by strain M. Expansion and survival of secondary memory CD8^+^ T cells depends not only on autocrine IL-2 [47], but also on CD4^+^ T cell help via IL-2 secretion and promotion of CD25 on CD8^+^ T cells [48], [49]. As *Mtb* strains made not differences in CD25 expression on CD8^+^ T cells it is tempting to speculate that reduced CD8^+^ CTL maturation by strain M could be due to low endogenous IL-2 and to deficient cooperation of CD4^+^ T cells and not to a deficient signaling though IL-2 receptor.

The role of IL-2 signaling has been demonstrated in the regulation of perforin and granzyme gene expression in CD8^+^ T cells independent of its effect on survival and proliferation [50], in delayed production of granzyme B in IL-2 deficient CD8^+^ T cells, perforin expression and strong cytolytic function [47]. Consistently, we showed that addition of exogenous IL-2 increased the expression of lytic molecules and also of CD107 in *Mtb*-stimulated CD8^+^ T. Remarkably, in the presence of IL-2, M-stimulated CD8^+^ T cells expressed perforin and CD107 at similar levels than did cells stimulated with strains 410 or H37Rv, emphasizing the role of IL-2 in CD8^+^ CTL response. However, as IL-2 did not overcome granulysin and granzyme B contents in M-stimulated cells, other cytokines could be involved in M-induced CTL response.

In summary, strain M induces dysfunctional CD8^+^ CTL though the combination of subtle changes in CD69 signaling driving to low autocrine IL-2 secretion and impaired CD4^+^ T cell help through IL-2 secretion. However, impaired CD69 and IL-2 expression did not alter other CD8 functions [11], [51], suggesting qualitatively differences in memory CD8 T cell response induced by strain M. Ongoing studies are actually being performed to determine if structural differences between strains M and 410 could explain the different ability of these strains to evoke full functional CD8^+^ CTL. Finally, the reduced CTL activity could be considered as part of an evasion mechanism employed by the MDR strain M to leave the bacterial niche intact allowing the persistence and its successful spreading to the community.

## Supporting Information

Figure S1 **M-stimulated CD8+ T cells showed low loss of granzyme B expression upon exposure to autologous macrophages.** PBMC from 5 PPD^+^ healthy individuals were cultured alone (Control Ly) or with *Mtb* strains (H37Rv-Ly, M-Ly and 410-Ly) for 6 d and then co-cultured for further 4 h with non-stimulated (Ly+control-Mac) or *Mtb*-stimulated (Ly+*Mtb*-Mac) autologous macrophages. Cells were stained for surface CD3 and CD8 and intracellular perforin expression and evaluated by FACS. Representative dot plots are shown and upper right panels show the percentage of perforin^+^ cells in the CD8 subset (Granzyme B^+^/CD8).
(TIF)

Figure S2 **M-stimulated CD8+ T cells showed low loss of granzyme B expression upon exposure to autologous macrophages.** PBMC from 5 PPD^+^ healthy individuals were cultured as in Figure S1 and then cells were stained for surface CD3 and CD8 and intracellular granzyme B expression and evaluated by FACS. Representative dot plots are shown and upper right panels show the percentage of granzyme B^+^ cells in the CD8 subset (Granzyme B^+^/CD8).
(TIF)

Figure S3 **M-stimulated CD8+ T cells showed low loss of granulysin expression upon exposure to autologous macrophages.** PBMC from 5 PPD^+^ healthy individuals were cultured as in Figure S1 and cells were stained for surface CD3 and CD8 and intracellular granulysin expression and evaluated by FACS. Representative dot plots are shown and upper right panels show the percentage of granulysin^+^ cells in the CD8 subset (Granulysin^+^/CD8).
(TIF)
