# Supplementary figures and images for: Mycobacterium tuberculosis Multidrug Resistant Strain M Induces an Altered Activation of Cytotoxic CD8+ T Cells
Source: PLoS One. 2014 May 16;9(5):e97837. doi: 10.1371/journal.pone.0097837 (PMC4024032; doi:10.1371/journal.pone.0097837)

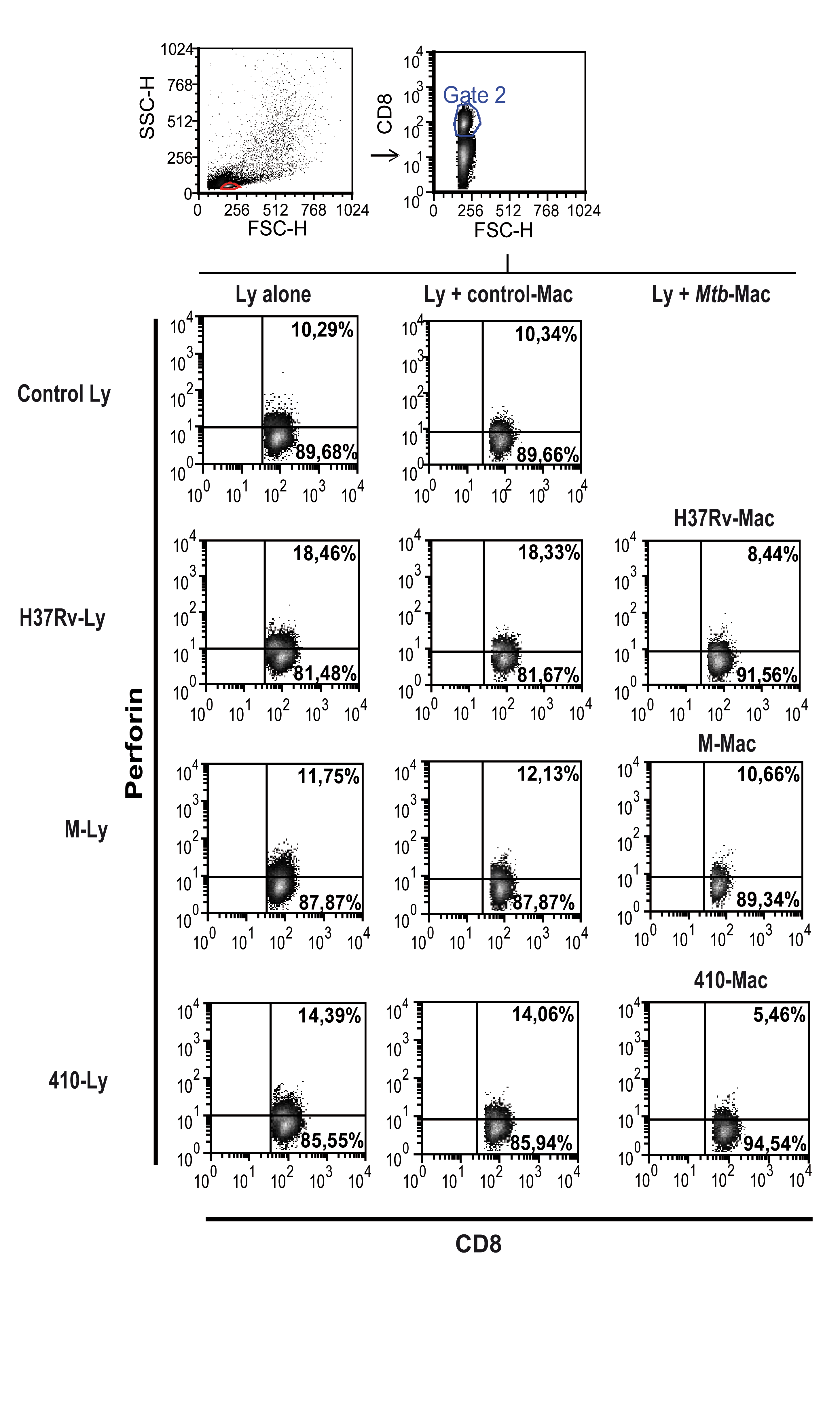

Supplement: Figure S1 — M-stimulated CD8+ T cells showed low loss of granzyme B expression upon exposure to autologous macrophages. PBMC from 5 PPD+ healthy individuals were cultured alone (Control Ly) or with Mtb strains (H37Rv-Ly, M-Ly and 410-Ly) for 6 d and then co-cultured for further 4 h with non-stimulated (Ly+control-Mac) or Mtb-stimulated (Ly+Mtb-Mac) autologous macrophages. Cells were stained for surface CD3 and CD8 and intracellular perforin expression and evaluated by FACS. Representative dot plots are shown and upper right panels show the percentage of perforin+ cells in the CD8 subset (Granzyme B+/CD8). (TIF) [file pone.0097837.s001.tif]

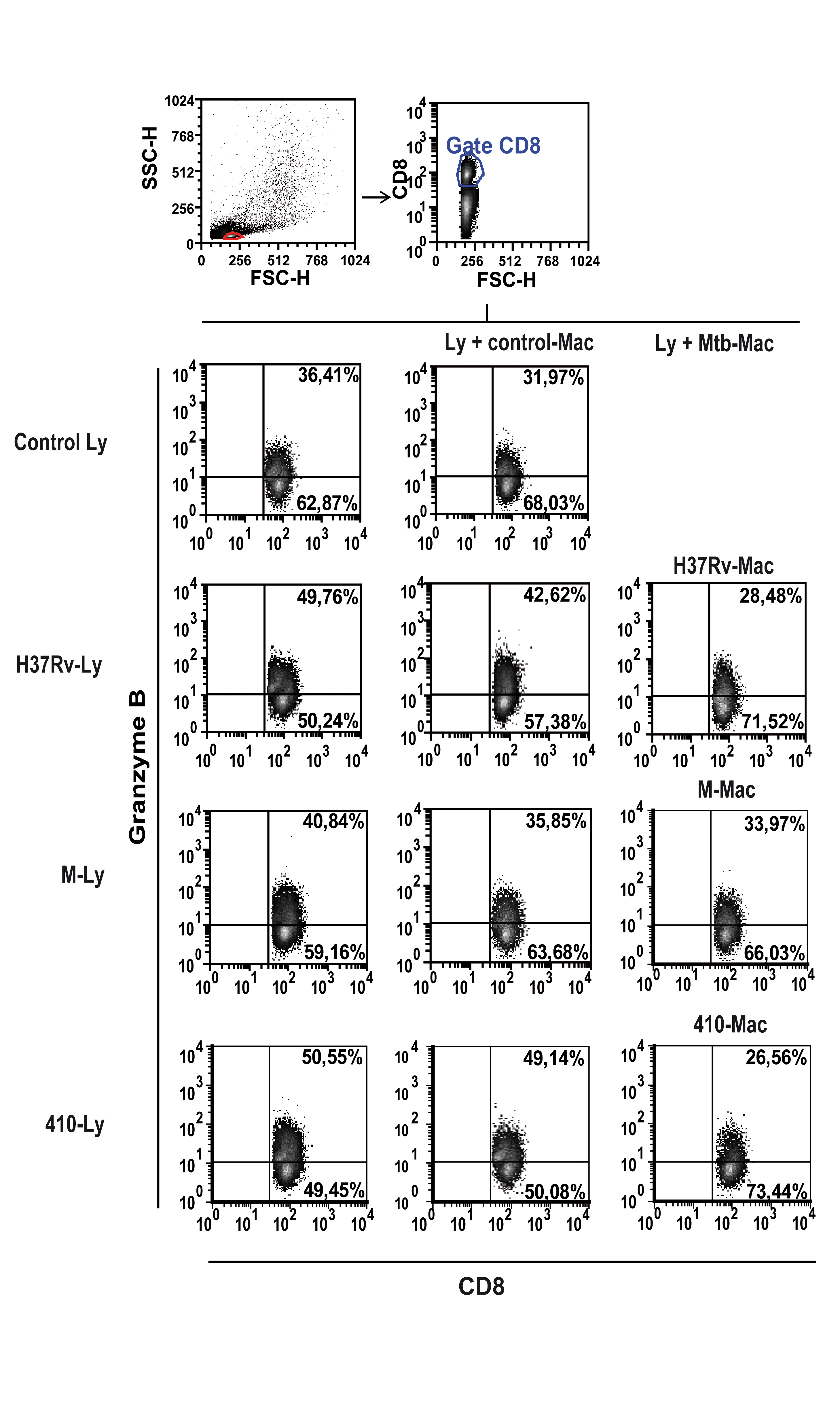

Supplement: Figure S2 — M-stimulated CD8+ T cells showed low loss of granzyme B expression upon exposure to autologous macrophages. PBMC from 5 PPD+ healthy individuals were cultured as in Figure S1 and then cells were stained for surface CD3 and CD8 and intracellular granzyme B expression and evaluated by FACS. Representative dot plots are shown and upper right panels show the percentage of granzyme B+ cells in the CD8 subset (Granzyme B+/CD8). (TIF) [file pone.0097837.s002.tif]

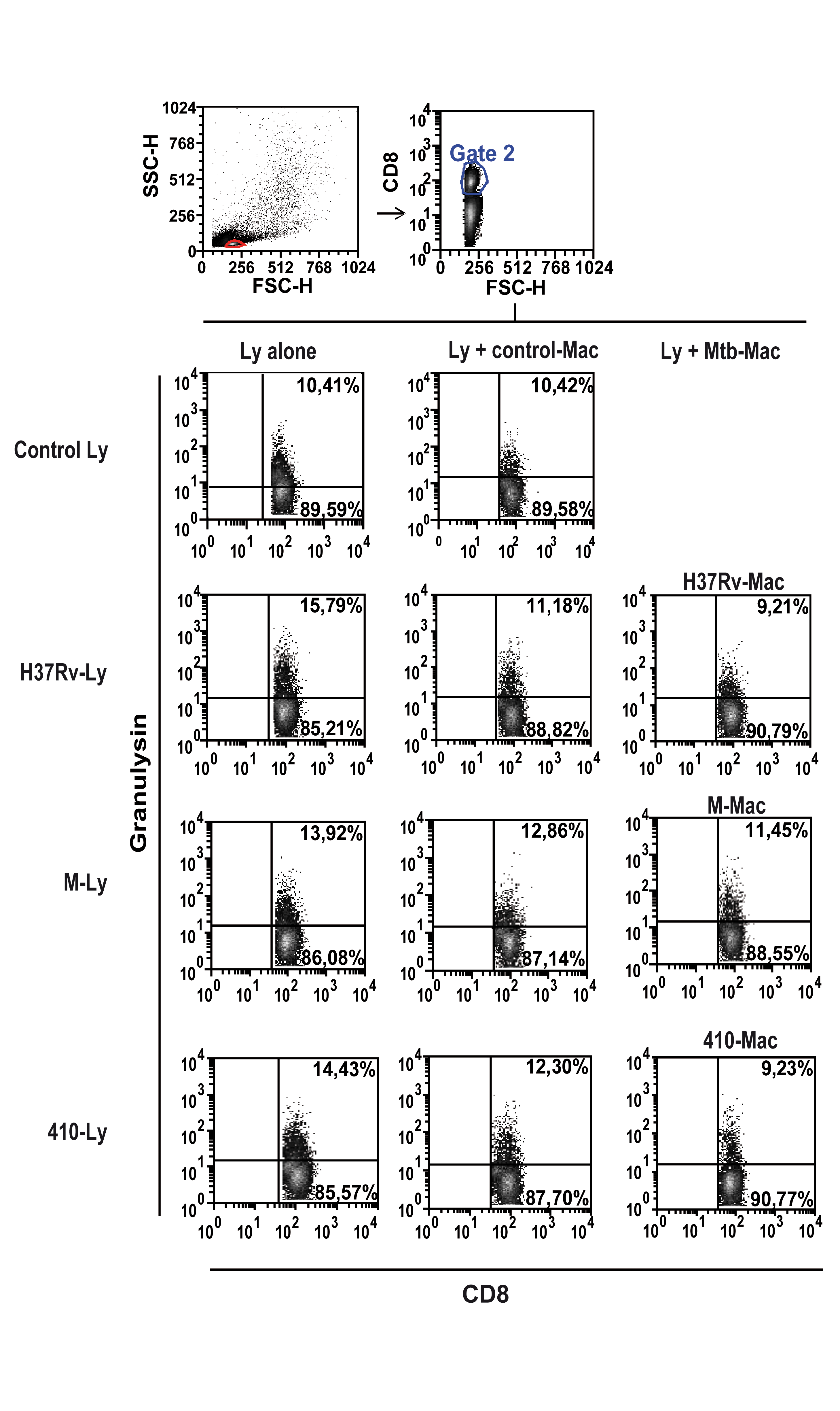

Supplement: Figure S3 — M-stimulated CD8+ T cells showed low loss of granulysin expression upon exposure to autologous macrophages. PBMC from 5 PPD+ healthy individuals were cultured as in Figure S1 and cells were stained for surface CD3 and CD8 and intracellular granulysin expression and evaluated by FACS. Representative dot plots are shown and upper right panels show the percentage of granulysin+ cells in the CD8 subset (Granulysin+/CD8). (TIF) [file pone.0097837.s003.tif]
